# Integration of Data from Liquid–Liquid Phase Separation Databases Highlights Concentration and Dosage Sensitivity of LLPS Drivers

**DOI:** 10.3390/ijms22063017

**Published:** 2021-03-16

**Authors:** Nazanin Farahi, Tamas Lazar, Shoshana J. Wodak, Peter Tompa, Rita Pancsa

**Affiliations:** 1 VIB-VUB Center for Structural Biology, Flemish Institute for Biotechnology, 1050 Brussels, Belgium; Nazanin.Farahi@vub.be (N.F.); Tamas.Lazar@vub.be (T.L.); Shoshana.wodak@gmail.com (S.J.W.); 2 Structural Biology Brussels, Vrije Universiteit Brussel, 1050 Brussels, Belgium; 3 Department of Biology, Technical University of Kaiserslautern, 67663 Kaiserslautern, Germany; 4 Institute of Enzymology, Research Centre for Natural Sciences, 1117 Budapest, Hungary

**Keywords:** liquid–liquid phase separation, liquid demixing, membraneless organelles, dosage sensitivity, protein abundance, quantitative proteomics, local concentration, data integration

## Abstract

Liquid–liquid phase separation (LLPS) is a molecular process that leads to the formation of membraneless organelles, representing functionally specialized liquid-like cellular condensates formed by proteins and nucleic acids. Integrating the data on LLPS-associated proteins from dedicated databases revealed only modest agreement between them and yielded a high-confidence dataset of 89 human LLPS drivers. Analysis of the supporting evidence for our dataset uncovered a systematic and potentially concerning difference between protein concentrations used in a good fraction of the in vitro LLPS experiments, a key parameter that governs the phase behavior, and the proteomics-derived cellular abundance levels of the corresponding proteins. Closer scrutiny of the underlying experimental data enabled us to offer a sound rationale for this systematic difference, which draws on our current understanding of the cellular organization of the proteome and the LLPS process. In support of this rationale, we find that genes coding for our human LLPS drivers tend to be dosage-sensitive, suggesting that their cellular availability is tightly regulated to preserve their functional role in direct or indirect relation to condensate formation. Our analysis offers guideposts for increasing agreement between in vitro and in vivo studies, probing the roles of proteins in LLPS.

## 1. Introduction

An important recent discovery in the field of molecular cell biology is that the formation of biomolecular condensates in living cells is driven by a reversible process called liquid–liquid phase separation (LLPS) [1]. These condensates, the so-called membraneless organelles (MLOs), represent distinct liquid phases selectively enriched in certain macromolecules and fulfill essential cellular functions under normal conditions and in response to stress [2,3,4,5]. Nucleoli, stress granules, P-bodies, germ granules, postsynaptic densities, heterochromatin, and many other long-known or recently discovered cellular compartments that belong to this category [6] have been reported in organisms from all kingdoms of life [7,8]. The functional benefits of MLOs do not directly derive from the individual role of their constituent molecules but emerge from their collective behavior [3,4,9,10,11,12,13]. Therefore, this recently recognized process is now considered as a fundamental mechanism employed by living cells to cost-efficiently [5] organize and reorganize cellular space and material according to functional needs [3,14,15,16].

A number of studies have demonstrated that MLOs exhibit fluid-like dynamics and general behavior [17,18,19]. In contrast to classical organelles, MLOs are reversible supramolecular assemblies that enable thermodynamically driven exchange of material with the surrounding solvent [20]. It has also been observed that, in perturbed cellular states, phase-separated liquid-like structures can transition into less dynamic hydrogels or solid-like protein aggregates [21,22,23,24]. The latter often contain long filaments resembling amyloid fibers, involved in many neurodegenerative diseases [25] such as amyotrophic lateral sclerosis [26,27], frontotemporal dementia [28], and Alzheimer’s disease [29,30,31], drawing attention to the potential pathological roles of liquid condensates.

LLPS is a complex and ill-understood process driven by multivalent weak interactions [20]. It has a very heterogeneous molecular background, both in terms of the interacting macromolecular modules and the contributing molecular driving forces [2,3,32,33]. In addition, protein–protein and protein–nucleic acid interactions can both play a role in scaffolding the condensates. Protein–protein assemblies are driven by interactions between diverse protein modules, such as homo-oligomerization by domains or coiled-coil regions, interactions between intrinsically disordered regions (IDRs), domain-domain, domain-motif, or post-translational modification (PTM)-controlled molecular interactions [33]. The associated protein–nucleic acid interactions are also diverse, with RNA/DNA length- and sequence-specific [34], as well as structure-specific [34,35] interactions playing a role, mediated by well-folded domains (such as RNA-recognition motifs or KH domains) [36] or IDRs (such as RGG boxes [37,38]) of the participating proteins. At the atomic level, electrostatics, hydrophobic interactions, cation–π or π–π interactions, or a combination of those, are reported to play key roles [2,3,32,33,39,40]. 

The ability to undergo LLPS may be a property of many macromolecules under specific conditions, many of which may not be encountered in living cells. Therefore, only a subset of proteins will phase separate to form MLOs under physiologically relevant conditions [3]. Importantly, LLPS is particularly sensitive to environmental conditions, such as ionic strength, temperature, pH, crowding, or the concentrations of the participating macromolecules [3,41]. Therefore, the reversible formation of condensates often acts as an ultrasensitive mechanism for sensing subtle changes in the intracellular milieu [42] or as a buffering process, enabling the maintenance of fine-tuned intracellular concentrations of their constituent macromolecules [3]. Their sensitivity of LLPS enables sophisticated regulatory mechanisms via cellular parameters, such as pH, temperature, ATP, or ion concentrations. Regulatory mechanisms controlling the availability of competitive binders, PTMs, or alternatively spliced forms of the condensate components are likewise common [41,43,44,45]. 

To categorize a protein as “phase separating”, therefore, requires a system-level understanding of the phase diagram of the process in the cell, and the influence of cellular parameters and states thereof. However, such analyses remain extremely challenging because relevant key parameters are either not known or cannot be controlled. Instead, researchers turn to investigate LLPS in the test tube, where conditions can be readily controlled. There is, however, no guarantee that the findings of in vitro experiments accurately represent the process in living cells, where additional molecular species may be present and various regulatory mechanisms may be at play. It is, therefore, crucial that in vitro observations on condensate formation be confirmed by suitable in vivo experiments.

The flurry of publications reporting on new, experimentally verified cases of LLPS, and the mounting interest in the LLPS process created the motivation to develop dedicated databases. These include databases such as NsortDB [46], MSGP [47], and RNA granule database [48] that are limited to certain species or MLOs and include proteins based solely on evidence of localization to those MLOs, irrespective of their potential role in LLPS. Of much wider scope are four LLPS-dedicated databases: PhaSePro [14], LLPSDB [49], DrLLPS [50], and PhaSepDB [51], reported in the 2020 Database issue of Nucleic Acids Research. These databases curate data from the literature and aim to provide rich annotations on the LLPS process from all studied species and MLOs. Most of them also annotate the role(s) of proteins involved, thereby offering the scientific community data that can be integrated with information on protein sequence, 3D structures, and functional annotations, and used in various bioinformatics analyses. While the general focus of the four databases and the types of published studies that they curated are very similar, the data they store and the annotations they provide differ substantially (see References [52,53] for their detailed comparison). This is not too surprising considering the inherent complexity of LLPS in cells and the ensuing challenges of extracting meaningful and consistent information from the literature on the underlying molecular players and conditions.

Here, we evaluate these differences and examine their origins by analyzing the supporting evidence archived in the 4 wider-scope databases in light of established definitions for the four major LLPS-related protein categories (LLPS driver, co-driver, regulator, and client), and the underpinning experimental approaches typically used in LLPS studies. Building on this analysis, we derive a high-confidence dataset of human driver proteins whose central role in LLPS is sufficiently supported by physiologically relevant in vivo and in vitro experiments. Given the key role protein concentration plays in controlling the LLPS process, attention is then devoted to rationalizing the data on protein concentrations used in the supporting experiments and linking the findings to the requirement for evolution to fine-tune the cellular availability of LLPS driver proteins in order to preserve their functional role in direct or indirect relation to LLPS formation.

We hope that our consolidated dataset of human LLPS proteins will inspire other systematic analyses of the available data on LLPS, highlighting further factors that need to be taken into account when designing, interpreting, or judging the biological relevance of LLPS experiments.

## 2. Results and Discussion

### 2.1. Interpretation of LLPS Experiments to Define the Roles of Proteins in the Formation and Integrity of MLOs

Due to the heterogeneity of the underlying driving forces, molecular mechanisms, and macromolecules contributing to LLPS, comprehensively elucidating functional-regulatory details of LLPS systems is a highly challenging task. Experimental approaches to phase-separating systems have been thoroughly reviewed recently [3,19]. Here, we rather focus on the interpretation of the results with regards to the functional roles of LLPS-related proteins, as outlined below.

In principle, a broad range of techniques has been applied and adapted for detecting and/or characterizing LLPS. As our purpose here is to clarify distinct roles of phase-separating proteins and understand the reasons for substantial differences between data in the four LLPS-related databases, we first clarify some cornerstone concepts that are not always articulated in LLPS studies.

(1) Strictly speaking, the capacity to phase separate is not a binary classifier, i.e., not the intrinsic property, of a protein, rather a contextual property of the protein and its environment (temperature, pH, partners, etc.). For considering a protein “phase-separating”, this behavior has to be approached under “native” or “native-like” conditions that incorporate all important constituents.

(2) Proteins have distinct roles (types of contributions) in phase separation, which may even differ between conditions in the test tube and the cell. Roughly, we may distinguish drivers (scaffolds), co-drivers (co-scaffolds), regulators, and clients, as defined below. There are various different experimental approaches that contribute to identifying the role of a given protein in LLPS.

(3) Phase separation depends on the concentration of the protein (among other parameters), i.e., practically any protein can be made to phase separate at sufficiently high concentrations if the environmental conditions allow it. From the biological (i.e., not polymer-chemical) point of view, we should accept a protein as phase separating if it does phase separate at concentrations compatible with physiological (or pathological) conditions.

(4) LLPS is not the equivalent of biomolecular condensation in general. Whereas LLPS is the process of demixing that leads to the formation of dense liquid droplets, condensation is a much broader category that encompasses all reactions of physical assembly, also including gelation, crystallization, clustering, polymerization, and amorphous or amyloid aggregation.

As a rule of thumb, we may state that for a complete and correct classification of a given protein with regards to the role (if any) it plays in LLPS, integration of multiple experimental approaches is necessary. We should appreciate that each approach provides different and often complementary information, i.e., in a sense they all have “advantages” and “disadvantages”. In general, the major advantage of in vitro experiments is that the components of the system are known and they can be perfectly controlled, whereas their disadvantage is that conditions are over-simplified and cannot accurately recapitulate physiological conditions (in terms of partners, post-translational modifications, metabolites, cellular crowding, etc.). On the other hand, the major advantage of in vivo measurements is that they do report on the LLPS behavior under genuine physiological conditions (unless protein(s) are severely overexpressed), ensuring the biological relevance of the LLPS process. Their major disadvantage resides in the largely hidden underlying cellular complexity because key parameters that determine or influence the LLPS process are either unknown or cannot be controlled.

In general, LLPS systems can only be adequately explored, the underlying molecular mechanisms fully uncovered and the roles of the components precisely determined, if in vivo and in vitro experiments are used in combination and the liquid material state of the resulting condensates is verified. In the following, we define the major categories of LLPS-related proteins on the basis of the distinct roles they play in the LLPS process. For each category, we provide a short “operational” description of the experimental evidence required to ascertain them.

**Driver (scaffold)** is a protein able to phase separate on its own (given appropriate, native-like conditions) to a dense liquid droplet, without the need for other macromolecular partner(s). We consider “driver” and “scaffold” as synonymous. If the presence of RNA is mandatory for LLPS, both the protein and RNA are considered as “co-drivers” (see next section).

In vitro, a driver protein is observed to phase separate when conditions (such as temperature, concentration, PTM state, the presence of a crowder, etc.) are right. In this framework, small molecules facilitating the LLPS of a driver (e.g., particular buffer, salt, metabolite) are considered as “conditions”, and not as molecular partners in the LLPS process. In vivo, overexpression of a driver, its oligomerization (driven by a PTM or optogenetics), or translocation to another organelle is sufficient to cause LLPS, i.e., the appearance of cellular puncta (membraneless organelles). Physiological cellular/local concentrations are preferable, and disappearance of the organelle upon deletion/silencing of the protein is not necessarily conclusive for its driver role.

**Co-driver** is a macromolecule (protein, RNA or DNA) that strictly requires another co-driver for phase separation. The two (or more) co-drivers are usually equally mandatory for LLPS (e.g., partners in signalosomes built on domain–motif interactions), however, in some cases, one co-driver can phase separate on its own at very high concentrations and its partner is required to lower its saturation concentration to physiologically relevant levels (e.g., RNA in some ribonucleoprotein droplets). A co-driver has a different role from a “regulator”, which also promotes the LLPS of its partner, but it does not physically take part in LLPS, i.e., will not be part of the resulting MLO scaffold. Most LLPS-centric databases analyzed here do not distinguish between drivers and co-drivers, therefore in the rest of the article, when we refer to drivers, we actually mean an umbrella term that covers both drivers and co-drivers.

In vitro, co-driver activity is apparent if there are more than one interacting macromolecules strictly required for LLPS or when LLPS of one co-driver occurs at a much lower (physiological) concentration in the presence of the other co-driver. In vivo, information on the co-localization of the co-drivers in the same puncta is essential (as often RNA is part of RNP particles).

**Regulator:** Often, the LLPS of a driver or co-drivers requires the presence (activity or activation) of an additional protein, which does not physically take part in condensate scaffold formation, though. There are a variety of regulators, such as modifying enzymes, transport proteins regulating cellular localization, or transcription factors promoting the expression of the driver and/or co-driver.

Although not frequently addressed in vitro, one may think of a kinase as a regulator catalyzing a critical phosphorylation event required for the phase separation of the target protein. In vivo, knocking out or silencing a gene may reveal a regulator, if its product has a profound (positive or negative) effect on LLPS but does not need to be part of the core assembly of driver/co-driver molecules that constitute the scaffold of condensates.

A **client** is not required for and does not have an effect on LLPS (unlike a co-driver or regulator), but it can localize to the formed condensate (through direct or indirect interaction with the driver or co-driver).

This behavior can be demonstrated both in vitro and in vivo by co-localization of the client with the drivers/scaffolds inducing LLPS. In in vitro partitioning experiments, the ability of proteins to enter the condensates already (pre-)formed by other proteins is tested. Importantly, the ability to enter pre-formed condensates can confirm the role as a client, but not as a driver/co-driver. 

### 2.2. Four LLPS Databases

In the following, we briefly outline the main characteristics of the four wider-scope LLPS databases (DBs), whose data are used to derive a consolidated dataset of human proteins that act as drivers/scaffolds in liquid–liquid phase separation processes. 

PhaSepDB is a comprehensive resource storing the proteins only on the basis of localization to MLOs [51]. The DB stores information on a total of 2957 proteins. It accepts three different evidence types for protein localization: literature evidence, UniProt localization annotations, and the results of high-throughput protein localization experiments. Consequently, PhaSepDB does not categorize the proteins by their role in LLPS. 

LLPSDB stores almost 1200 in vitro LLPS experiments as entries, addressing the LLPS behavior of 273 proteins [49]. These comprise natural proteins as well as artificially designed protein constructs. The DB provides detailed information on the molecular components and measurement parameters for each experiment, along with their outcomes (e.g., LLPS is detected or not). There is no attempt at interpreting experimental data and/or defining the role of proteins in LLPS.

DrLLPS classifies LLPS-related proteins into scaffolds, regulators, and clients as assessed from related literature quotes processed by automated text mining, followed by curator assessment [50]. The underlying literature evidence mostly reports on high-throughput and low-throughput experiments addressing the physical or functional association of proteins with MLOs (for clients), phenotypic effects of their knockout, silencing, or overexpression on MLOs (for regulators) as well as on dedicated LLPS experiments (for scaffolds). The DB also includes proteins in different organisms predicted to phase-separate by homology transfer (based on sequence homology with proteins experimentally shown to do so). 

PhaSePro stores a relatively small, manually curated set of 121 LLPS proteins as entries, all categorized as ‘drivers’ based on experimental evidence from in vivo and/or in vitro studies [14]. Rigorous curation criteria are used to categorize a protein as an LLPS driver, which takes into account the physiological relevance of conditions reported for the associated experiments. PhaSePro entries also contain information on additional determinants and regulators of the LLPS process as well as on the associated molecular mechanisms.

While two of the four investigated DBs rely on localization/association evidence (PhaSepDB completely, DrLLPS partly), the other two solely rely on dedicated LLPS experiments (LLPSDB and PhaSePro). With regards to the assignment of the specific roles of proteins in the LLPS process, DrLLPS is the only database classifying proteins into distinct categories according to their role in phase separation (scaffolds/regulators/clients). PhaSePro evaluates these roles in the curation process, but only annotates drivers as entries. In LLPSDB, which is entirely dedicated to in vitro experiments, the role of the protein in LLPS is not explicitly assigned but may be derived from the annotations associated with individual experiments. PhaSepDB contains no information on the role of the protein in phase separation.

### 2.3. Data Consolidation across LLPS Databases Reveals Inconsistencies in Protein Annotations

A useful means for assessing the quality and consistency of the data stored in different databases covering a common field of research is to compare equivalent data items across the DBs, evaluate the level of consensus (overlap) and complementarity (differences) in these items, and identify the origins of detected discrepancies [54,55]. We followed this strategy for the four LLPS-dedicated databases. To be able to compare equivalent subsets of the proteins, we restricted our comparison to LLPS drivers/scaffolds, which are considered synonymous and involve both self-sufficient drivers and co-drivers.

**Figure 1:**
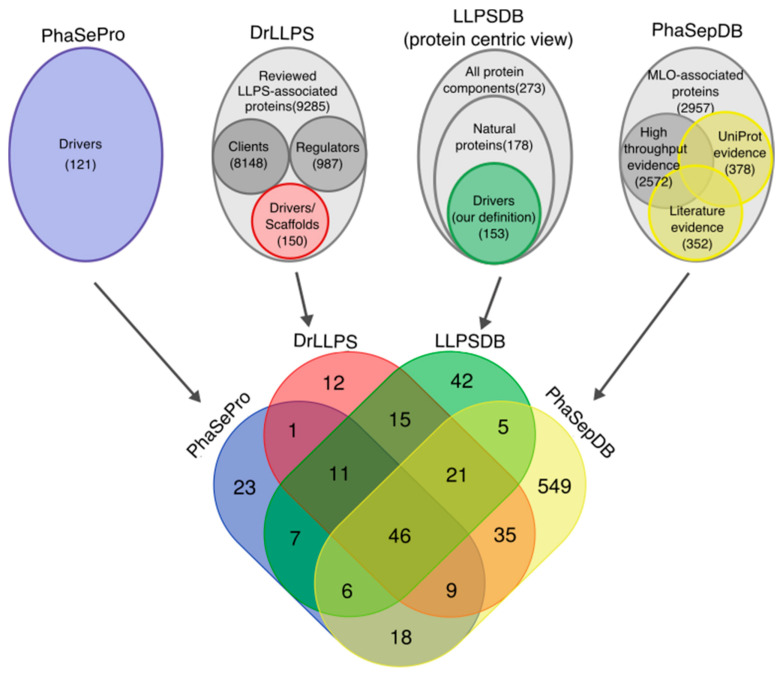
Content and overlap of the four liquid–liquid phase separation (LLPS) datasets. The contents of the four databases (PhaSePro, DrLLPS, LLPSDB, PhaSepDB) are represented in the upper portion, with subsets of the data used in this analysis highlighted in color, whereas the rest of their data are depicted in different shades of gray. The total number of proteins as well as the number of proteins in the different subsets are indicated. In the Venn diagram at the bottom, the overlap of the colored subsets of the four LLPS databases is shown.

We collected 121, and 151 driver proteins, corresponding, respectively, to all entries in PhaSePro and to entries labeled as ‘reviewed scaffolds’ in DrLLPS. Extracting this information reliably from LLPSDB and PhaSepDB, which do not explicitly annotate protein roles, was less straightforward and involved applying various specific filters to the curated data (see Section 3.1, and Figure 1). Using these filters on the annotations of the in vitro experiments in LLPSDB, we extracted 153 native proteins that fulfilled our criteria of LLPS drivers at least regarding partner-dependencies. A much larger set of 689 MLO-associated proteins potentially acting as drivers was extracted from PhaSepDB on the basis of literature evidence and UniProt localization annotation (Figure 1).

The overlap of the collected driver proteins across the 4 DBs is in general quite poor (Figure 1). In total, only 46 driver proteins are shared by all 4 DBs. PhaSePro, DrLLPS, and LLPSDB, taken pairwise, share on average ~54% of their driver proteins. Although the 689 potential driver proteins from PhaSepDB covers on average ~63% of the drivers from each of the other databases, it shares only a small fraction (20%, or 140 proteins) with all three databases taken together. This suggests that this much larger set of PhaSepDB proteins probably contains many non-driver proteins (clients and regulators), which could not be eliminated because the database does not compile information on the precise role of proteins in LLPS. We, therefore, limit our analysis to the remaining three databases, where this information is provided.

**Figure 2:**
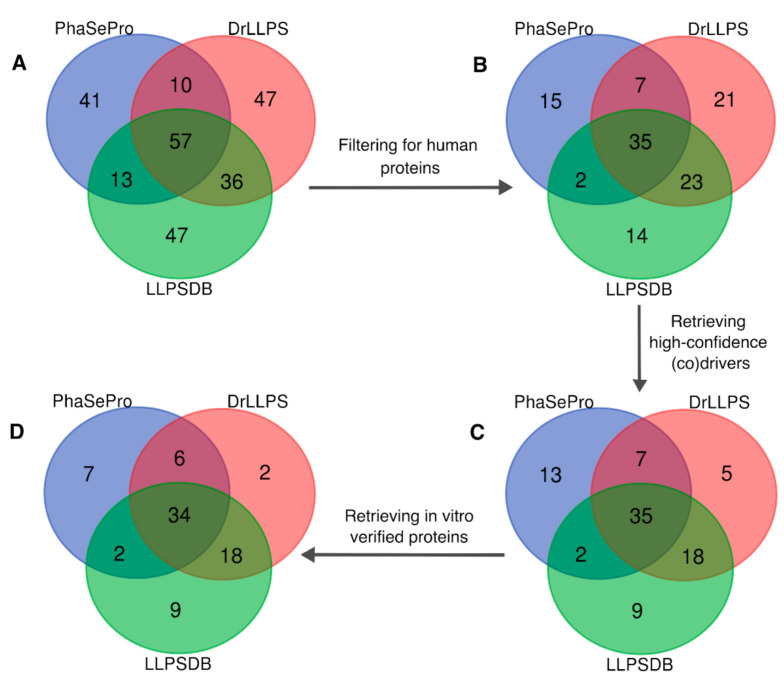
Filtering steps for obtaining a consolidated set of high-confidence human LLPS drivers. (**A**) Venn diagram representing the overlap of the three LLPS databases (251 proteins from PhaSePro, DrLLPS, and LLPSDB) after the exclusion of data in PhaSepDB. (**B**) Venn diagram representing the same overlap after filtering for human proteins (117 proteins). (**C**) Human proteins were further filtered for high-confidence (co)drivers by excluding those that could not be accepted as LLPS (co)drivers according to our definition of this role, resulting in a set of 89 proteins. (**D**) Among these, we identified 78 in vitro verified proteins, which could be used in the concentration analysis.

Figure 2A and Supplementary Table S1 depict the overlap of the driver proteins collected from PhaSePro, DrLLPS, and LLPSDB. A total of 57 driver proteins are shared by all 3 DBs, representing <50% of the proteins annotated as drivers in any of them. A larger number of such proteins are shared by pairs of databases (93 for DrLLPS/LLPSDB, 70 for PhaSePro/LLPSDB, and 67 for PhaSePro/DrLLPS). On the other hand, as many as 135 driver proteins are unique to one of the three DBs with 41, 47, and 47, such proteins found in PhaSePro, DrLLPS, and LLPSDB, respectively.

Such discrepancies between annotations in databases are not uncommon and may have a number of origins. They may result from: (1) differences in the literature that is being covered; (2) differences in curation policies (extracting information from abstracts or full publications, with or without consulting figures and supplementary information); (3) making different choices about the information to be archived; (4) differences in the interpretation of published information, which need to be understood in order to improve and standardize curation practices.

With regards to literature coverage, we find that each of the 3 databases curated a unique set of publications not covered by the other two DBs (Supplementary Figure S1). The number of unique publications covered by PhaSePro, DrLLPS, and LLPSDB represents 17.8%, 12.4%, and 28.7% of the total number of publications covered by all three databases (202), respectively. The fraction of unique publications is the highest for LLPSDB due to its experiment-centric rather than protein-centric approach, resulting in highly studied proteins, such as RNA-binding protein FUS, to be supported by multiple articles. Different policies on archiving supporting publications (e.g., selectively or more comprehensively), further contribute to the observed discrepancies (see legend of Supplementary Figure S1B, for details). Closer scrutiny also reveals that most of the 41 LLPS drivers unique to PhaSePro, such as those encoded by genes *TIAR-2*, *MORC3*, *U2AF2*, *MATR3*, and others are derived from articles published during the summer of 2019, too recent to be considered by the analyzed first releases of the other two databases. 

Another source of the detected differences concerns alternative co-driver proteins that can act as substitutes for each other in the same LLPS system. This is for instance the case for, the proteins Gads (*GRAP2* gene product) and SLP76 (*LCP2* gene product), identified as alternatives to Grb2 and Sos1, respectively, in the LAT signalosome [56], the many R-motif containing proteins identified as possible alternative co-drivers acting together with nucleophosmin [57], or the androgen receptor, identified as an alternative of Daxx in driving phase separation with Speckle-type POZ protein (*SPOP* gene product) [58]. These alternative co-drivers are mentioned in the entry pages of the respective systems in PhaSePro but are not included as independent entries in the database, a direct consequence of the current schema design of this database.

Different curation policies are another source of discrepancies. For instance, PhaSePro only focuses on native proteins reported as LLPS drivers in experiments carried out under physiological conditions, whereas DrLLPS and LLPSDB are less strict in this regard. For example, the E3 SUMO-protein ligase PIAS2 and tyrosine kinase ABL1 are classified as drivers in DrLLPS and LLPSDB, although the supporting publications report experiments carried out with protein constructs containing 10 tandem copies of the SUMO-interacting motif of PIAS2, or 4 tandem copies of the proline-rich region of ABL1 [48], which introduce artificial, non-physiological multivalency into the studied systems. PIAS2 and ABL1 are not archived in PhaSePro, because the native protein chains were not shown to undergo LLPS [59]. 

Other examples include the yeast RNA-binding protein Mip6 and human γ-D-crystallins. Mip6 is classified as a scaffold protein only in DrLLPS, based on a report that it undergoes LLPS in vivo when highly overexpressed [60]. The assignment of γ-D-crystallins as LLPS drivers by LLPSDB is supported by evidence that rat and human γ-D-crystallins undergo LLPS under high pressure/low-temperature conditions, which are only relevant for proteins of deep-sea organisms [61]. The non-physiological conditions in which these proteins are reported to undergo LLPS may be relevant for certain cellular pathologies, but they do not support classifying these proteins as LLPS drivers under normal physiological conditions (see driver definition in Results Section 2.1).

Lastly, an important more fundamental reason for the limited overlap of LLPS drivers in the different databases stems from inconsistencies in interpreting experimental data by database curators. For example, DrLLPS curators appear to employ more lenient criteria for categorizing proteins as scaffolds compared to those by PhaSePro for drivers, which, in principle, are equivalent categories. For instance, among the entries unique to DrLLPS, we found several proteins (products of genes *CIRBP*, *CPEB2*, *RBM3*, and others) that are not demonstrated to undergo LLPS on their own or with a well-defined set of co-drivers. These proteins partition into condensates formed by other proteins [62,63], which defines them as clients but not drivers (cf. Results Section 2.1). 

In other instances, DrLLPS annotated proteins as scaffolds (such as the products of genes *G3BP1*, *RBFOX1*, *LSM4*, *pgl-1*, and others), but had borderline evidence only. If their driver roles were also supported by PhaSePro, they were annotated as candidate entries. While the central role of the products of genes *G3BP1* and *pgl-1* in the formation of the respective liquid-like MLOs (stress granules and *C. elegans* P-granules, respectively) is irrefutable, these proteins were not shown to undergo LLPS in vitro and, therefore, their partner dependencies are not (yet) sufficiently elucidated.

Another reason for doubting the driver role assigned to some proteins may lie with the physical properties of the resulting assemblies. For example, the proteins encoded by the human *RBFOX1* [64] and yeast *LSM4* [65] genes were not shown to form liquid-like droplets but were found to assemble into fibrous aggregates of irregular shapes in in vitro experiments, and were, therefore, not classified as LLPS drivers in PhaSePro (see Section 2.1 on evidence supporting the liquid material state of condensates). Some of these divergent approaches to the interpretation of the experimental data stem from the complexity of the analyzed systems and hinge more on curation policies related to this complexity.

### 2.4. A Consolidated Dataset of Human LLPS Driver Proteins

In total, 251 LLPS driver proteins were retrieved from the three resources (Figure 2A). From this set, we extracted 117 human driver proteins (Figure 2B), representing the largest number of driver proteins from a single organism. The latter set was pruned for proteins (28 in all) that could not be accepted as LLPS (co)drivers/scaffolds according to our definition of this role (see Section 2.1); many of these were mentioned in the previous section. This yielded a consolidated set of 89 human LLPS driver proteins (Figure 2C and Supplementary Table S2), whose physiologically relevant form was studied under physiologically relevant conditions in experiments that support LLPS (co)driver roles and where the liquid state of the condensates was also confirmed. The full list of gene names of the excluded proteins (together with reasons of exclusion) is detailed in Methods Section 3.2.

The resulting consolidated dataset of 89 human LLPS driver proteins should be useful for future studies analyzing different aspects of LLPS and provides an excellent opportunity to further interrogate the underlying data.

### 2.5. Concentrations of LLPS Driver Proteins

Protein concentration, that of driver proteins, in particular, is a crucial parameter of condensate formation that governs the phase diagrams of the underlying process. While protein concentrations are only approximately adjusted and imprecisely quantified when probing LLPS inside cells, they can be precisely controlled in in vitro LLPS studies. However, the main challenge of these studies is to define the physiologically relevant range of protein concentrations to be sampled. For lack of better data on the cellular organization of the proteome, in vitro studies often refer to whole-cell protein concentrations derived from proteomics studies to define this range, even though, as our analysis will be showing, these cellular concentrations are often not the ones governing the LLPS process [27,55,56,57,58,59].

To find out more on how this challenge is handled in the field, we focus on the subset of 78 proteins from our consolidated dataset of human LLPS divers, with available in vitro LLPS measurements and concentration values (Figure 2D). Next, we compare the concentrations of these proteins used in the in vitro LLPS experiments to their cellular abundance levels, measured in mass spectrometry (MS)-based quantitative proteomics studies, obtained from PaxDb [66]. The data on protein concentrations used in the corresponding in vitro studies were obtained from LLPSDB and from the literature, with special attention to collecting values for the saturation concentration C_sat_ (the lowest protein concentration giving rise to LLPS), whenever possible (see Methods Section 3.3, for details and Supplementary Table S3 for the assembled dataset). Tissue-specific and cell-line integrated abundance values retrieved from PaxDb, available for 75 proteins of the consolidated subset, were converted to concentrations using a published formula [67] and plotted for the same set of proteins for comparison (see Methods Section 3.3 for details, and Supplementary Table S4 for the derived concentration values). 

**Figure 3:**
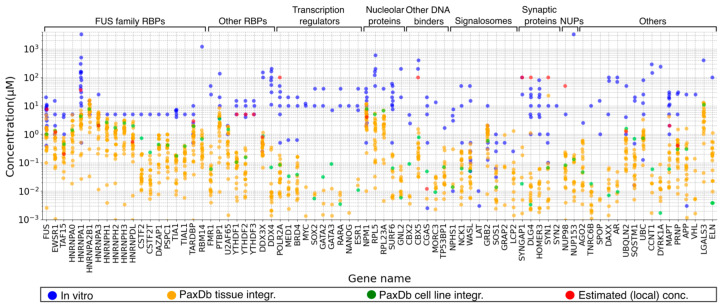
Protein concentrations applied in in vitro LLPS experiments frequently exceed those calculated from proteomics-derived cellular protein abundances. Comparison of protein concentrations used in in vitro LLPS experiments (in blue), concentrations calculated from proteomics-derived cellular protein abundances stored in the PaxDb database (tissue-specific integrated values in orange and cell-line integrated values in green), and local concentrations estimated or referred to by authors (in red) for human LLPS driver proteins integrated from the three LLPS resources and filtered for in vitro verified true LLPS (co)drivers. Concentrations are provided in micromolar units, while LLPS proteins are represented by the respective gene names on the horizontal axis. Functional groups of the proteins are indicated on the top of the figure. For the products of genes *LAT*, *SYN2*, and *YTHDF3*, PaxDb concentration values were not available.

The results of this comparison, depicted in Figure 3, show that protein concentrations used in in vitro LLPS experiments are in many cases considerably higher, often by one or two orders of magnitude, than cellular concentrations in different tissues or the corresponding value integrated across different human cell lines. The values for the two types of concentration in Figure 3 tend to display a significant spread, with some particularly high in vitro values reported for proteins, such as those encoded by the genes *RBM14* [68], *NUP153* [69], *CCNT1*, and *DYRK1A* [70], where the authors used lyophilized protein powders and provided the applied amounts as mg/mL. Considering that only the lowest protein concentration (e.g., C_sat_) at which a protein undergoes LLPS can be used to deduce that the observed process is physiologically relevant, we decided to compare only the lowest in vitro concentration where a given protein was observed to phase separate (ideally, C_sat_) to the highest of its PaxDb-derived cellular concentrations. The latter choice is legitimate since many of these proteins are expressed at detectable levels in only one or a few specific tissues or cell types. 

This second comparison (see Supplementary Figure S2) reveals that only 25 of the 75 proteins (33.3%) could undergo LLPS in vitro at a concentration that is within the range of its PaxDb-derived cellular concentrations (their lowest in vitro concentration is lower than the highest PaxDb value). For the remaining proteins, the lowest in vitro concentration was higher than the corresponding highest PaxDb concentration by less than (17 proteins; 22.7%) or more than (33 proteins; 44%) an order of magnitude.

However, for 30 of these proteins, it was not possible to relate the reported concentrations to the LLPS C_sat_ values, as data on the concentration-dependent in vitro LLPS phase diagram was lacking (see red gene names in Figure S2), making it difficult to reach any conclusion.

Some of these issues may affect the observed differences. However, the magnitude and systematic nature of these differences suggest that other factors are at play. In the following, we, therefore, examine some of these factors. We discuss their possible implications with regards to the in vitro LLPS studies, the current estimates of protein cellular concentrations, and the organization of the cellular proteome.

### 2.6. Protein Concentrations: An Elusive Parameter in Both In Vitro and In Vivo LLPS Studies

#### 2.6.1. Uncertainties Associated with Current Cellular Protein Abundance Measures

First, we note that the cellular protein abundance values obtained from PaxDb are derived from quantitative proteomics studies that measure the number of protein copies per cell. The values provided by the database represent the relative number of protein copies expressed in units of parts per million and are converted to protein concentrations using published estimates of the number of protein molecules/fl of cellular volume in model organisms, including human, and the average estimated cell volume [67] (see Methods for detail). In addition, it is well documented that proteomics methods are much better at quantifying highly expressed proteins than those expressed at low levels. The relative copy numbers (or cellular concentrations) of lowly expressed proteins, therefore, tend to be underestimated [71,72,73,74]. The quantification of membrane proteins is also quite poor due to solubility issues [75]. Solubility issues may also hamper accurate detection/quantification of proteins that are part of supramolecular complexes or condensates [76], although the latter possibility still needs to be explored. 

**Figure 4:**
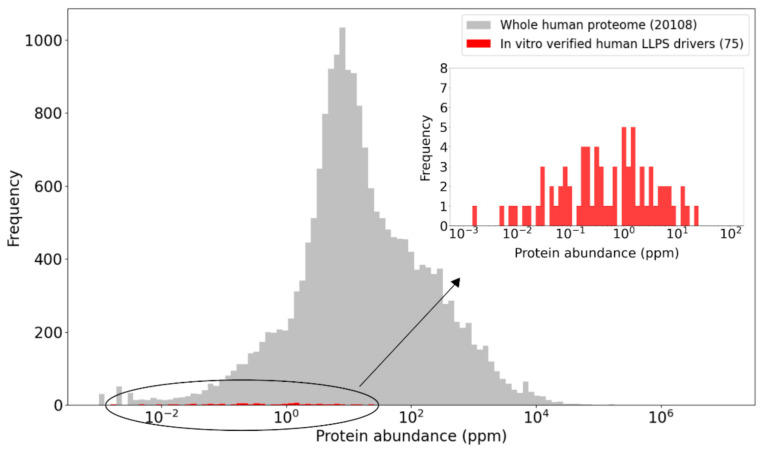
Human LLPS driver proteins are of relatively low abundance compared to the proteome average. The highest tissue- or cell type-specific integrated abundance value was derived for each human protein from PaxDb. These maximal abundances of human LLPS driver proteins (red) are compared to those of the proteome (grey) using histograms. The abundances of LLPS drivers are also separately depicted in the inset histogram to ensure better resolution of the data. Abundances are provided in parts per million (ppm) units on the X-axis.

These limitations likely directly lead to the underestimation of measured cellular concentrations of the human LLPS driver proteins in Figure 3, in line with the observation that they tend to be low abundance proteins, as illustrated in Figure 4. This is not surprising as many of the proteins driving LLPS belong to protein families of typically low abundance, such as signaling proteins [73], transcription factors [74,77], chromatin-associated proteins [78], or postsynaptic density proteins [79,80], which are at the detection limits of current proteomics studies.

#### 2.6.2. Local Concentrations of LLPS Driver Proteins in Cellular Niches Define Their Phase Diagrams, but What about Measuring Them?

Most proteins are not evenly distributed within cells but are localized to specific cellular niches, which define the specific biochemical environment and sets of interaction partners, required to carry out their function [81]. While a number of mass spectrometry-based techniques, referred to as spatial proteomics, are able to reliably assign the subcellular localizations of thousands of proteins and confirm the above tendency, their ability to quantify protein copy numbers in specific localization is still limited [82,83]. Many, if not, most LLPS-driver proteins are known to display highly restricted patterns of subcellular localization, often due to local translation of their mRNA [76,82,83,84,85,86,87,88] and/or anchoring to larger molecular building blocks in the cell [89,90,91,92,93,94,95,96]. This suggests that the context-dependent local concentration of these proteins, rather than their overall cellular concentration, is the parameter that determines the phase diagram of the corresponding in vivo LLPS process. This assumption seems to be the premise for using measures [97,98,99,100,101,102,103] or estimates [92,95,104,105] of in vivo local protein concentrations to define the protein concentration range used in a sizable fraction of the in vitro studies. However, it is not always clear what they represent: do they refer to the concentrations inside the condensates or do they also include the surrounding local ‘bulk’ proteins? In either case, they are expected to be significantly higher than the corresponding PaxDb values.

Among our studied LLPS driver proteins, local translation reportedly applies to postsynaptic-density (PSD) proteins in nerve terminals, such as PSD-95 (gene *DLG4*), SynGAP (gene *SYNGAP1*), Homer-3 (*HOMER3*), and Synapsin-1 (gene *SYN1*), which reach local concentrations of ~100 μM in quantitative proteomics studies of purified postsynaptic densities and nerve terminals [76,86,87]. These high local concentrations justify the use of the respective in vitro LLPS concentrations [10,102] over those of the much lower PaxDb-derived cellular concentrations (1.1 μM, 0.35 μM, and 0.062 μM for PSD-95, SynGAP, and Homer-3, respectively). Examples of very high local in vivo concentrations of LLPS drivers due to being specifically anchored to repetitive binding sites on larger, slowly-diffusing macromolecular entities in the cell (such as RNA, DNA, or membranes), include the RNA polymerase II subunit encoded by gene *POLR2A*, for which all PaxDb-derived concentrations are below 0.2 μM. The corresponding in vitro LLPS study uses protein concentrations of 0.5–50 μM [92], citing evidence that its local concentration in the nucleus is estimated to reach ~1 μM, which is further increased by several orders of magnitude locally, at the sites of transcription [106]. Other examples include the hU2AF65 splicing factor [89], YTHDF proteins [90], Erα [94], FG-rich nucleoporins (NUPs) [95], and the heterochromatin protein HP1 alpha (gene *CBX5*) [107].

Some LLPS studies may use even higher protein concentrations to induce phase separation than the local in vivo concentrations would warrant, because of failing to adequately reproduce the cellular context. For example, the presence of even low amounts of crowding agents remarkably boosts most LLPS processes studied in vitro [39,108,109]. However, in vitro experiments sometimes poorly match cellular crowding conditions exerted by the totality of cellular macromolecules [110], requiring as a result the use of higher protein concentration to observe condensate formation [110]. In other cases, such as for the nucleoporins, in vitro LLPS experiments often probe the behavior of one representative of a larger ‘family’ of functionally related proteins [69] that contribute to the in vivo LLPS process [95], requiring here too the use of higher concentration of the representative to compensate for the absence of other family members. High in vitro protein concentrations could also be required to compensate for the lack of right partners that could promote a more physiological-like LLPS process. For instance, in the case of transcription factors and co-activators, very high protein concentrations were required to achieve in vitro LLPS initially [63,111], but recently it was shown that the presence of DNA fragments harboring multiple copies of the specific recognition elements of the transcription factors largely lower their saturation concentrations [96]. Similarly, chromatin marks and important protein partners were recently discovered to contribute to the LLPS of HP1 proteins [107,112] and remarkably lower their saturation concentrations [113].

While recognizing the challenge of evaluating in vivo local protein concentrations, the above considerations offer a valid rationale for using measures or estimates of these ‘effective’ local concentrations as a yardstick for in vitro LLPS studies, rather than the proteomics-derived cellular concentrations as often proposed [39,99,108,114,115,116]. Our analysis also underscores the importance of validating the findings of in vitro LLPS studies by carefully crafted in vivo experiments inside cells, and vice-versa. This is the case for most of the proteins in our consolidated high confidence dataset of 89 human LLPS divers and it is also the case for most of the proteins (109/121) stored in PhaSePro. The essential role of these proteins in LLPS can, therefore, be considered as well supported by the available data, as further evidenced by the analysis of the dosage sensitivity of the genes coding for these proteins presented below.

### 2.7. Dosage Sensitivity of LLPS Driver Genes

Considering that protein concentration is a crucial determinant of the highly cooperative LLPS process [57,117,118,119], and assuming that liquid condensate formation is an important functional property of LLPS drivers, their availability in the cell would need to be tightly regulated. It has in fact been suggested that proteins with the ability to trigger LLPS may become toxic upon increased expression, suggesting a potential link of dosage sensitivity to disease [60]. Furthermore, it is well established that LLPS-associated proteins are enriched in IDRs prone to engage in promiscuous interactions, properties that also require tight regulation of cellular concentrations of proteins [120] and dosage sensitivity of the corresponding genes [121]. Gene dosage is defined as the copy number of a particular gene in a genome, and dosage sensitivity is the measure of intolerance to modifications of gene dosage. So far, the possible relationship of LLPS-associated proteins with dosage sensitivity was limited to a comparison of predicted physicochemical properties (disorder, RNA-binding, amino acid composition) of proteins that become toxic upon overexpression in yeast with those that localize to granules [60].

Here, we use our consolidated dataset of human LLPS drivers to clarify the potential relationship of LLPS and dosage sensitivity. To this end, we evaluate the extent to which our 89 human LLPS drivers (Figure 2C) are over- or under-represented in the sets of the most reliable dosage-sensitive (MRDS) and most reliable dosage-insensitive (MRDIS) human genes recently consolidated [122].

**Figure 5:**
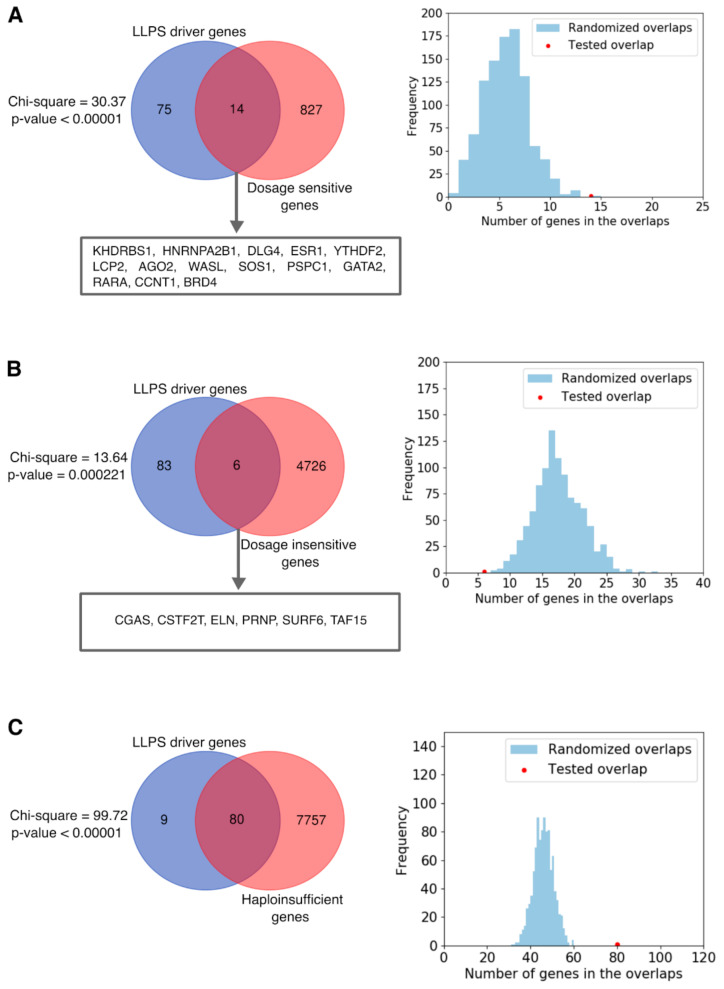
The genes of human LLPS drivers are overrepresented in dosage-sensitive genes. The Venn diagrams (left) show the overlap between human LLPS driver genes and the set of most reliable dosage-sensitive (MRDS) genes (**A**), the set of most reliable dosage insensitive (MRDIS) genes (**B**), and the set of haploinsufficient genes (**C**). The genes in the intersections are listed for (**A**,**B**). The detected overlaps represent statistically highly significant enrichments (**A**,**C**) or depletion (**B**) based on chi^2^ tests using the whole human-reviewed UniProt proteome as background. Histograms (right) showing the difference between the overlaps of human LLPS-associated genes (red) or equivalent sets of randomly selected well-annotated genes (blue) with (**A**) MRDS, (**B**) MRDIS, and (**C**) haploinsufficient genes.

We found a strong enrichment of human LLPS-associated genes in MRDS genes (chi^2^ test, *p* < 0.00001) (Figure 5A) and a strong depletion in MRDIS genes (chi^2^ test, *p* = 0.000221) (Figure 5B) using the reviewed human proteome from UniProt [123] as a background. We noted that human LLPS proteins are generally very well annotated, in comparison to many non-LLPS proteins. To avoid bias from the different levels of annotation among human proteins, we repeated the analysis by using randomized selections of similarly well-annotated subsets of the human proteome as a background. LLPS-associated genes displayed a much higher overlap with MRDS genes and a much smaller overlap with MRDIS genes than any of the equivalent 1000 random gene sets, providing solid statistical evidence for their dosage sensitivity (Figure 5A,B).

The set of dosage-sensitive genes [122] was consolidated from 4 main sources, comprising 2 datasets of haploinsufficient genes [124,125], and one dataset each of ohnologs [126] and copy number-conserved genes [127], which display different flavors of dosage sensitivity. Haploinsufficiency measures the intolerance to heterozygous loss of function (when protein products of one of the two alleles are lost) [122,124]. Ohnologs are pairs of genes originating from whole-genome duplications [126]. If a gene has only ohnologs but no paralogs, or shows conserved copy numbers across mammalian genomes, it is also considered dosage-sensitive [122]. To find out which of these properties contribute to the detected dosage sensitivity of LLPS-associated genes more, we retrieved the above-mentioned 4 original datasets, mapped them to UniProt, integrated the two datasets of haploinsufficient genes, and performed the relevant enrichment analyses (for the integrated datasets, see Supplementary Table S5). We found that LLPS-associated genes are highly enriched in haploinsufficient genes (80/89 were found in the list of 7837 haploinsufficient genes) (Figure 5C, Supplementary Table S6). LLPS-associated genes were also enriched in ohnologs (Supplementary Figure S3A) but not in copy number-conserved genes (Supplementary Figure S3B) based on chi^2^ tests. These results were confirmed by analyses based on randomized selections (Figure 5C and Supplementary Figure S3, Supplementary Tables S6 and S7).

The MRDS dataset [122] is significantly enriched in transcription factors (TFs), which tend to be tightly regulated low-abundance proteins. To verify that the observed highly significant enrichment for haploinsufficient genes or ohnologs is not due to an enrichment of LLPS drivers for transcription factors, we evaluated the enrichment for TFs in the LLPS-associated dataset, using the same dataset of transcription factors as in Reference [122] (a list of 1639 TFs originally published in Reference [128]). The results showed no enrichment in TFs among the LLPS-associated genes (9 TFs of 89 genes; chi^2^ test, *p* = 0.456 using the reviewed human proteome as background), as well as no enrichments in TFs among the LLPS-associated genes that overlap with the MRDS, haploinsufficiency, or ohnologs datasets as shown by Supplementary Figure S4.

This analysis indicates that both losses and gains in the copy number of LLPS-associated genes may be deleterious to cells, i.e., their dosage sensitivity is not an exclusive consequence of the overexpression of the corresponding gene products as previous suggested [60], but reflects a deleterious perturbation of the cellular availability of LLPS-associated proteins, in line with their important roles. In our view, the propensity for dosage sensitivity reflecting a tight regulation of their availability lends strong support for their role in in vivo condensate formation, validating the results of associated in vitro LLPS experiments.

## 3. Methods

### 3.1. Analysis of the Overlap between LLPS Databases

In order to analyze the overlap between the 4 publicly available LLPS databases, we needed to make sure that we compare subsets of their data that are equivalent across the databases.

PhaSepDB is based on protein localization evidence (association to MLOs) and not on the ability to undergo LLPS, so it was not possible to filter for LLPS drivers. Therefore, only UniProt annotated and reviewed subsets of the database were obtained from PhaSepDB [51] (version 1.3, October 2019), and entries based on high-throughput evidence were regarded as less reliable and were not included.

From PhaSePro [14] (version 1.1.0), we obtained all the 121 entries.

DrLLPS [50] (version 1.0) is represented by its 150 ‘reviewed scaffold’ proteins in this analysis.

From LLPSDB [49], we only accepted natural proteins that have shown phase separation in at least one of the corresponding experiments. Since this database stores in vitro LLPS experiments as entries and does not categorize the investigated proteins according to their role played in LLPS, we needed to introduce a definition of drivers based on the associated supporting in vitro experiments. We accepted proteins as drivers that: (1) could undergo phase separation on their own, as one-component systems, or (2) could only undergo LLPS as essential components of two- or multi-component systems (with other proteins, DNA or RNA), wherein the rest of the components could not undergo LLPS on their own in another experiment. This filtering was necessary in order to make sure that proteins used as accessory components (like EGFP or mCherry) or regulators in the experiments are not accepted as drivers. In total, 153 proteins were obtained from this filtering protocol. We note, however, that this simple protocol, which considers only the dependence of condensate formation on the molecular partners and was applied automatically, is only a partial substitute for careful curation of the associated experiments, which would evaluate the physiological relevance of these experiments as a whole. 

Comparison of the entries of the databases (provided as Supplementary Table S1) was done based on UniProt ACs of the canonical proteins; information on isoforms (even where available) was omitted. It is important to mention that for some of the driver proteins the full-length protein has not been tested for LLPS in the supporting primary publications, where only smaller segments have been expressed and purified. These proteins are also accepted as drivers based on the assumption that if a segment of a protein undergoes LLPS then most likely the full protein will also be able to do so.

### 3.2. Consolidation of the Human LLPS Driver Protein Dataset

A set of 251 proteins was consolidated from three of the four LLPS databases: PhaSePro, DrLLPS, and LLPSDB. The fourth database, PhaSepDB, was excluded from the consolidation since it does not contain annotations on the role proteins play in LLPS, and the associated literature showed a poor overlap with those of the other three resources. This consolidated set was filtered for human proteins to yield 117 proteins.

Next, we excluded the following human proteins that did not correspond to LLPS (co)drivers/scaffolds according to our definition of this role (Results Section 2.1): Proteins under the gene names *ABL1*, *PIAS2*, *SUMO3*, *ITSN1*, and *C9orf72* were excluded because they only served as donors of protein modules amplified into artificial repeat proteins for LLPS experiments and the natural proteins have never been tested for LLPS (see associated experiments in LLPSDB). *CRYGD* and *EN2* were excluded because they were only tested for LLPS under highly non-physiological conditions (high pressure and/or low temperature). Proteins encoded by genes *TP53*, *KPNB1*, *KPNA2*, *FYN*, *MAP1LC3B*, *XPO1*, *RBM3*, *CPEB2*, *CIRBP*, *SGO1*, and *FBN1* were excluded because they were only shown to partition into the already formed condensates of other proteins (based on which they could be accepted as clients but not as (co)drivers) but were not demonstrated to undergo LLPS on their own or as necessary components of multicomponent LLPS systems (see associated experiments in LLPSDB and/or DrLLPS). *RBFOX1* was excluded because there is no evidence for the liquidity of the aggregates it formed [64]. *5HT1A* was excluded due to the respective study reporting on concentration units, particularly 1:19 w/v that we could not convert into µM [129]. Although *HNRNPAB*, *HNRNPA1L2*, and *HNRNPD* are present in LLPSDB, they were excluded because we could not see any droplets in the figure panels presenting their respective droplet formation experiments [39]. The above listed 23 proteins were excluded due to insufficient in vitro LLPS evidence, while further 5 proteins (namely, *ELAVL1*, *G3BP1*, *DYRK3*, *AXIN1*, and *ZNF207*) were excluded based on insufficient in vivo evidence (proteins supported by only in vivo evidence were accepted as drivers if both their localization to liquid foci and their necessity for the formation/integrity of the respective foci were proved).

The resulting set of 89 human proteins can be found in Supplementary Table S2.

### 3.3. Obtaining, Converting, and Comparing Protein Concentration and Cellular Abundance Values from Different Sources

We first obtained the protein concentrations applied in in vitro LLPS experiments (only those where LLPS was detected) either from LLPSDB (where available) or from the primary publications provided by the other resources. Where available, concentration values for the full-length wild type proteins were used, otherwise, concentrations of segments were also accepted. If there were multiple measurements with a positive LLPS outcome (e.g., with different measurement conditions, partners), we accepted all applied protein concentrations where a physiologically relevant form of the protein was studied (including simple truncates, modifications, mutations mimicking modifications) to obtain a distribution of values rather than a single data point. In cases when a range of concentration was sampled to produce a phase diagram [57,117,118,119], we recorded the average of the extreme values. Such averaging was performed for 86 of the 366 data points. In a subsequent manual curation round, these averaged values were replaced by the saturation concentrations (C_sat_) where available, with the latter being defined as the lowest protein concentration at which LLPS is observed. Concentration values were retrieved from information on phase diagrams in LLPSDB, or in the original supporting publications (see Supplementary Table S3). 

We accepted all conventional concentration units, like mg/mL, µM, nM, and converted them to µM. When converting units in mg/mL to µM, we accounted for the molecular mass of the protein construct used in the respective experiment, including truncated and fused (e.g., GFP) forms of the protein of interest, as well as sequence tags. We used the Compute pI/Mw tool of the ExPASy server (https://web.expasy.org/compute_pi/ accessed on 15 January 2021) to calculate the molecular weights of protein segments.

For the product of the *LAT* gene, concentrations were provided as molecules/µm2 as it is a membrane protein, however, the authors provided an estimation on what protein concentration this is equivalent to [56,66], so we accepted their estimate and used the same estimate in the case of nephrin (gene *NPHS1*), another membrane protein studied by the same research group.

Proteomics-derived protein abundance values were obtained from the integrated datasets of PaxDb, the Protein Abundance Database (version 4.1) [66]. To this end, UniProt ACs provided by the LLPS databases were mapped onto Ensembl protein IDs and abundance data available for the latter have been retrieved from PaxDb. If there were multiple available (tissue-specific or cell line integrated) abundance values for a protein, we accepted all to gain distribution of data rather than a single data point. The PaxDb abundance values reported in ppm (parts-per-million) as units were converted to micromolar concentrations using the below formula Equation (1) from [130], where *k ≈* 3·10^6^ proteins/fL, the Avogadro constant N_A_ = 6.02 × 10^23^ molecules/mol, and A is the abundance. This allowed comparisons with the in vitro applied LLPS protein concentrations retrieved from LLPSDB and the literature. For some of the gene products, like those of the *LAT* (transmembrane), *SYN2* (membrane-anchored), and *YTHDF3* genes, PaxDb did not have any abundance values, so we only show the concentrations reported for their in vitro LLPS experiments in the concentration graphs without a comparison.

C = (k · A) / N_A_ (1)

The publications reporting on the in vitro LLPS experiments performed for human LLPS proteins were screened for author statements referring to published or calculated estimates of the physiologically relevant cellular or local concentrations of the investigated proteins. These concentrations were collected (where available) and used as reference values besides PaxDb-derived abundance-based concentrations. For all the obtained concentration values depicted in Figure 3 grouped by genes and data sources, see Supplementary Table S4.

For the abundance comparison depicted in Figure 4**,** the highest tissue- or cell line-specific integrated PaxDb value was obtained for each human protein with such PaxDb data in ppm (approximately the full proteome), and their frequency distribution was used as a reference to be compared to the highest abundances of LLPS driver proteins.

### 3.4. Dosage Sensitivity Enrichment Analyses

The lists of 853 most reliable dosage-sensitive (MRDS) and 5579 most reliable dosage-insensitive (MRDIS) human genes were downloaded from the Table S5 of Ni et al. [122]. The lists of 3230 (probability of being LoF-intolerant (pLI) > 0.9) and 7841 haploinsufficient human genes were obtained from Lek et al. [124] and Shihab et al. [125], respectively. A list of 7294 ohnologs was collected from Makino et al. [126], while a list of 7014 copy number-conserved genes was taken from Rice et al. [127]. The reviewed human proteome (20,359 proteins) was retrieved from UniProt release 2020_04 [123]. MRDS, MRDIS, haploinsufficient and copy number-conserved genes, as well as ohnologs were mapped against UniProt by using the provided Ensembl transcript/gene identifiers (where available) or gene names. Only those were retained in the datasets that could be mapped against the reviewed human proteome. After merging the two lists of UniProt ACs for haploinsufficient genes, we gained 841, 4732, 7837, 6865, and 6948 genes for the five properties, respectively. The obtained gene lists with the mapped UniProt ACs are provided as Supplementary Table S5. The overlaps of the 89 human LLPS drivers with the five gene sets were computed, and chi^2^ statistics were applied to address the statistical significance of the overlaps using the reviewed human proteome (20,359 proteins) as background.

Since >95% of our integrated human LLPS driver proteins are reviewed UniProt proteins with an annotation score of 5 of 5 and evidence at protein level, we filtered the reviewed human proteome of UniProt for similarly well-annotated proteins and used the resulting subset of 13,389 proteins as a background for randomized selections to avoid any biases stemming from the large differences in annotation levels of human proteins. A total of 1000 protein sets of equivalent size to the set of 89 human LLPS drivers were selected and their overlaps with MRDS, MRDIS, haploinsufficient, and copy number-conserved genes as well as ohnologs were compared to that of LLPS drivers. These data are provided as Supplementary Tables S6 (for LLPS drivers) and S7 (for equivalent randomly selected genes sets).

### 3.5. Data Analysis and Representation Software Tools

The obtained data were analyzed with custom-made python scripts (Python version 3.7.9). Venn diagrams were produced by the UGent Bioinformatics and Evolutionary Genomics group’s online tool (http://bioinformatics.psb.ugent.be/webtools/Venn/ accessed on 15 January 2021). The histograms and the concentration graphs were generated using Python’s seaborn (version 0.10.1), matplotlib.pyplot (version 3.3.2), and pandas (version 1.0.1) modules. 

## 4. Conclusions

Consolidating the data on the LLPS driver/scaffold proteins from 3 recently published databases dedicated to archiving information on proteins associated with the formation of liquid condensates in living cells enabled us to derive a high confidence dataset of 89 human LLPS driver proteins supported by physiologically relevant experiments. This data consolidation exercise allowed us to appreciate the difficulties that database curators face in assigning the role played by specific proteins in the LLPS process, which may be at least partially alleviated if guidelines such as those we and others [52,53] propose, would be systematically followed.

In-depth scrutiny of the data on protein concentrations used in the LLPS experiments supporting our high confidence dataset of human driver proteins laid the uncertainties associated with defining the physiologically meaningful ranges of this important parameter that governs condensate formation, and suggested how these uncertainties may be mitigated and ultimately abridged.

Endeavoring to explain our findings on the systematically higher concentrations reported in in vitro LLPS studies than those measured for whole cells derived from quantitative proteomics (Figure 3) revealed that this trend is mainly due to the fact that in vitro studies often strive at reproducing the cellular context, albeit only imperfectly. Given that most LLPS-driver proteins display highly restricted patterns of subcellular localization, where they may encounter and interact with other (often) unbeknown cellular components, in vitro studies employ measurements [97,98,99,100,101,102,103] or estimates [92,95,104,105] of protein concentrations in local cellular niches to design their experiments, which are indeed expected to be much higher than the corresponding PaxDb values. Although these estimates are often imprecise because effective niche concentrations cannot be reliably measured by current quantitative proteomics techniques [82,131], they still represent effective (local) concentrations much better than PaxDb-derived values. The latter values should, therefore, be considered as theoretical lower limits, but not as mandatory reference values for most in vitro LLPS studies. One should keep in mind, however, that while quantifying local protein concentrations within cells is currently not feasible, LLPS drivers may indeed display surprisingly high local protein concentrations despite their overall low cellular abundance.

Our analysis and its implications highlight that gaining a quantitative understanding of the proteome organization in living cells, and its implications for the formation of condensates and MLOs, is an important challenge that the phase separation field needs to address. Our findings that dosage-sensitive genes, haploinsufficient genes and ohnologs in particular, are overrepresented among human LLPS drivers, underscore furthermore the requirement of keeping the cellular abundance of the respective protein products at an optimal level compatible with tightly regulated LLPS behavior, to avoid serious pathologies that deviations in any direction may cause.

In conclusion, our analysis underscores the need of taking extreme care in designing and interpreting LLPS experiments, while considering the specificities of individual LLPS systems. Furthermore, the highly selective nature of membraneless organelles [11,65,95,132,133,134,135,136] implies that proteins are not only recruited to their specific niches but are also excluded from those where they do not belong. The continued discovery of novel MLOs should, therefore, transform our classical, stochastic picture of the cell interior to one that is more highly organized and spatially constrained.

## Data Availability

The authors confirm that the data supporting the findings of this study are available within the article and its supplementary materials. The program codes used for the filtering and integration of the data are available from the corresponding authors upon reasonable request.

## Supplementary Materials

The following are available online at https://www.mdpi.com/1422-0067/22/6/3017/s1, Figure S1: Overlap of the literature references addressing the investigated entries, Figure S2: The lowest protein concentrations applied in in vitro LLPS experiments frequently exceed those calculated from proteomics-derived highest cellular protein abundances, Figure S3: The genes of human LLPS driver proteins are overrepresented among ohnologs but not among copy number-conserved genes, Figure S4: Lack of enrichment in transcription factors among LLPS driver genes, Table S1: List of 251 potential LLPS drivers from all organisms (before filtering) derived from the different resources and depicted in Figure 2A, Table S2: List of 89 confident human LLPS drivers derived from the different resources (depicted in Figure 2C), Table S3: In vitro concentration data with sources and information on the protein constructs, Table S4: Sources of concentration data (in vitro, proteomics-derived and estimated) used in the analysis and depicted in Figure 3, Table S5: Dosage sensitivity features of human proteins from the reviewed human proteome of UniProt, Table S6: Dosage sensitivity features of the 89 confident LLPS drivers, Table S7: Dosage sensitivity features of genes in the randomly selected well-annotated gene sets.
