# Supplementary material for: Integration of Data from Liquid–Liquid Phase Separation Databases Highlights Concentration and Dosage Sensitivity of LLPS Drivers
Source: Int J Mol Sci. 2021 Mar 16;22(6):3017. doi: 10.3390/ijms22063017 (PMC8002189; doi:10.3390/ijms22063017)
Supplement: Supplementary file 1 [file ijms-22-03017-s001.zip › a_Farahi_supplementary_figures.pdf]

Supplementary materials to:

Integration of data from liquid-liquid phase separation databases highlights concentration and dosage sensitivity of LLPS drivers

Nazanin Farahi [1,2,3], Tamas Lazar [1,2], Shoshana J. Wodak [1,2], Peter Tompa [1,2,4], Rita Pancsa [4]

[1] VIB-VUB Center for Structural Biology, Flemish Institute for Biotechnology, Brussels, Belgium
[2] Structural Biology Brussels, Vrije Universiteit Brussel, Brussels, Belgium
[3] Department of Biology, Technical University of Kaiserslautern, Kaiserslautern, Germany
[4] Institute of Enzymology, Research Centre for Natural Sciences, Budapest, Hungary

Supplementary figures:

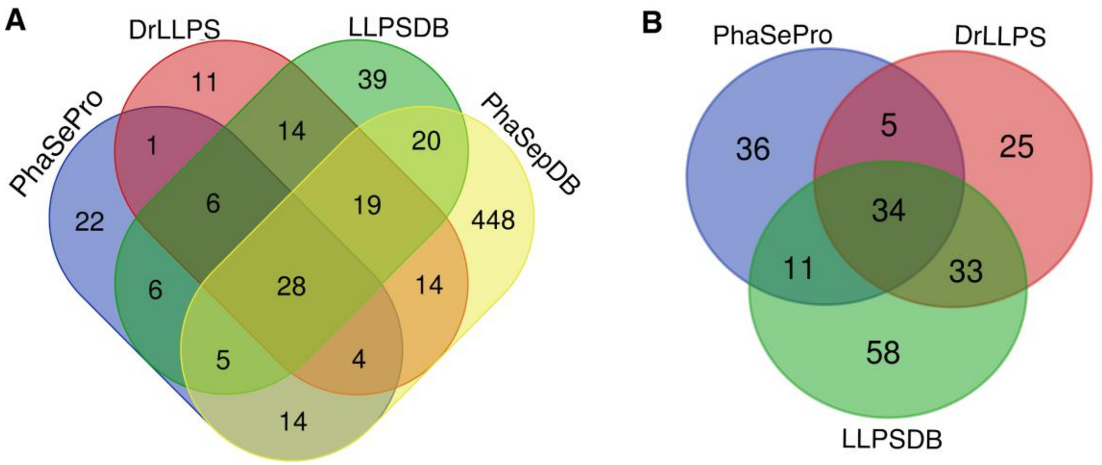

Supplementary Figure S1: Overlap of the literature references addressing the investigated entries. A) The Venn diagram compares the literature references supporting the localization to MLOs/ ability to undergo LLPS of the selected subsets of LLPS-associated proteins across the four resources. B) The same Venn diagram is shown for the three resources that could be analyzed further.

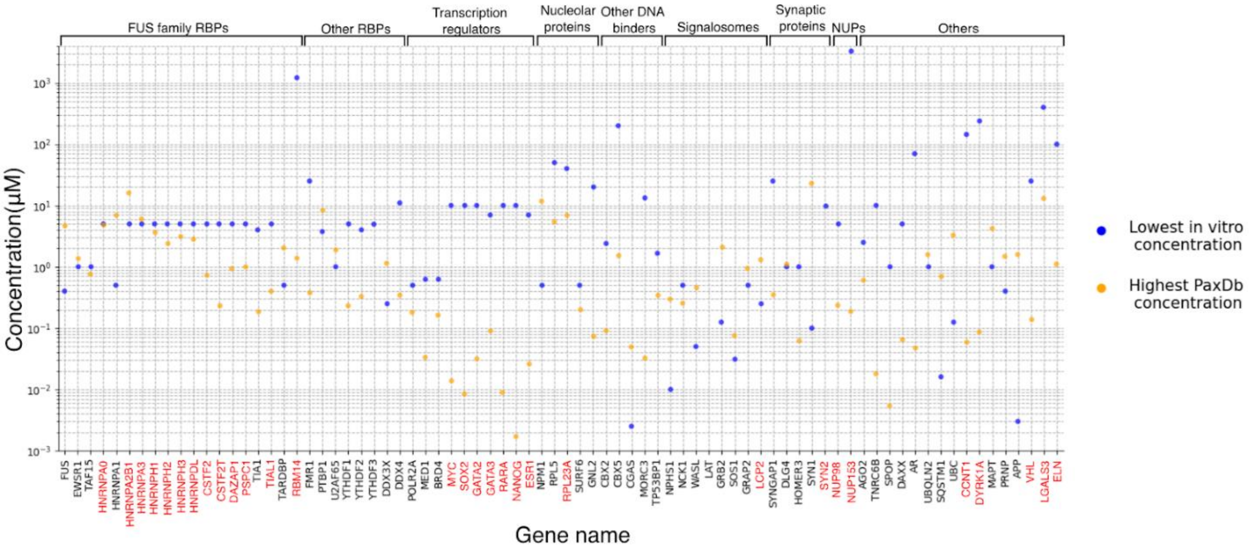

Supplementary Figure S2: Lowest protein concentrations applied in *in vitro* LLPS experiments frequently exceed those calculated from proteomics-derived highest cellular protein abundances. Comparison of the lowest protein concentrations applied in *in vitro* LLPS experiments where the given proteins could phase separate (in blue) to the respective cellular concentrations calculated from proteomics-derived highest protein abundances stored in PaxDb (in orange) for the 78 true human LLPS (co)driver proteins backed by *in vitro* experiments. Only integrated datasets were considered for PaxDb. Concentrations are provided in micromolar units, while LLPS proteins are represented by the respective gene names on the horizontal axis with red gene names marking proteins with no available saturation concentration. Functional groups of the proteins are indicated on the top of the figure. For 3 proteins, LAT, SYN2 and YTHDF3, PaxDb concentration values were not available.

25 of the 75 proteins (33.3%) depicted in Figure S2 could undergo LLPS *in vitro* at a concentration that is within the range of its PaxDb-derived cellular concentrations (their lowest *in-vitro* concentration is lower than the highest PaxDB value. A further 17 proteins (22.7%) could undergo LLPS *in vitro* at a concentration that is within one order of magnitude from its highest PaxDb-derived cellular concentration. The remaining 33 proteins (44%) could not undergo LLPS at close-to-cellular concentrations.

Adequately evaluating these differences is difficult because *in vitro* experiments were often carried out only at a single “comfortable” concentration. That is, no concentration-dependent phase diagram was reported for at least the following 30 proteins (represented by red gene names), therefore their saturation concentrations were not defined and thus their ability to undergo LLPS at physiologically relevant concentrations can hardly be judged. This partly explains the concentration deviations observed, since these proteins take up more than half of the groups where the applied concentrations show no overlap with the range of cellular concentrations (9/17 - 53% and 18/33 - 54.5%, respectively).

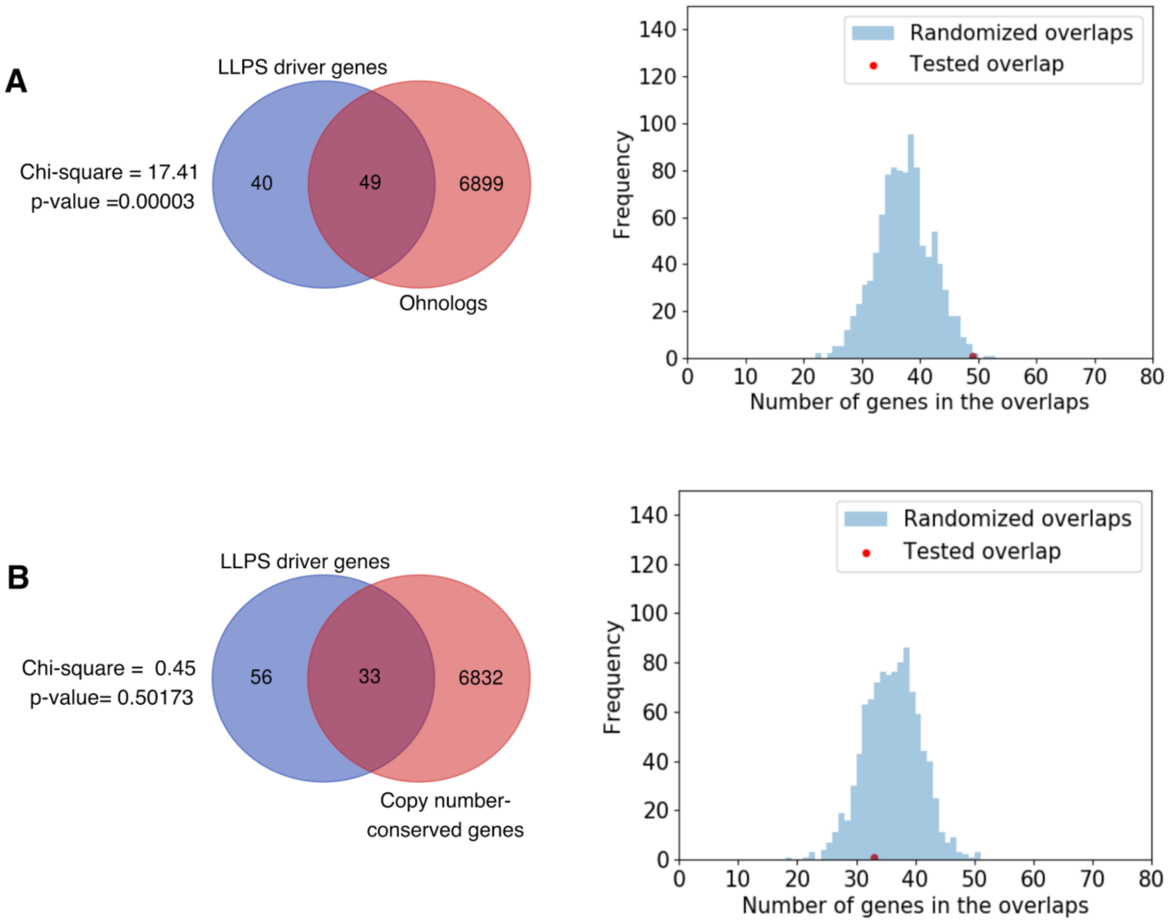

Supplementary Figure S3: The genes of human LLPS driver proteins are overrepresented among ohnologs but not among copy number-conserved genes. Enrichment analysis of human LLPS driver genes among (A) ohnologs and (B) copy number-conserved genes. The Venn diagrams show the overlap between human LLPS driver genes and (A) ohnologs or (B) copy number-conserved genes. The detected overlap is only significant for ohnologs based on chi^2 tests using the whole human reviewed UniProt proteome as background (left). Histograms showing the difference between the overlaps of human LLPS driver genes (red) or equivalent sets of randomly selected well-annotated genes (blue) with (A) ohnologs or (B) copy number-conserved genes.

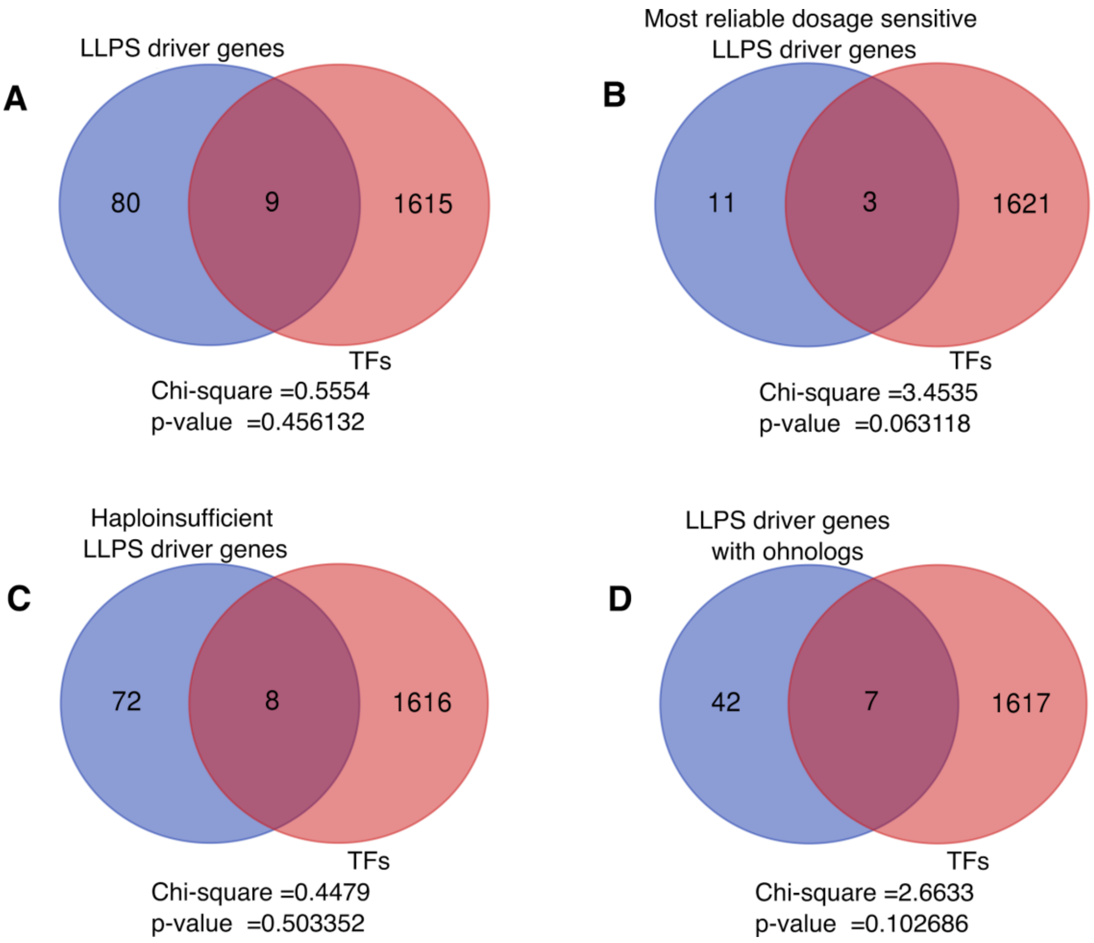

Supplementary Figure S4: Lack of enrichment in transcription factors among LLPS driver genes. The Venn diagrams show the overlap between the transcription factor (TF) genes and the human LLPS driver genes (A), the set of most reliable dosage sensitive (MRDS) LLPS driver genes (B), the set of haploinsufficient LLPS driver genes (C), and the set of LLPS driver genes with ohnologs (D). The detected overlaps do not represent statistically significant enrichments based on the chi^2 tests.
